# Poor-Law Nurses and the N.P.L.O.A.

**Published:** 1917-03-17

**Authors:** 


### POOR-LAW NURSES AND THE N.P.L.O.A.

### The Position Clearly Demonstrated.

*To the Editor of* The Hospital.

Sir,—My attention has been drawn to the amazing statements made by some of the speakers of the West Derby Union at the meeting of the N.P.L.O. Association at Liverpool regarding Poor-Law nurses and the College.

Misrepresentations and Absurdities.

They would be laughable if the matter was not so serious and the danger so evident. The picture of the Association holding out the right hand of fellowship to "their sister officers," who are being treated, according to some of the speakers, with scant courtesy and consideration by the College of Nursing, is highly amusing to those who know the inner workings of the Poor Law. After many years of studied neglect, the Association has suddenly woke up to the fact that the nurses are a large and increasingly important body in the Poor Law. It is therefore anxious to enrol them, nominally for their own good, but principally for the benefit of the Association. There are thousands of nurses in the Poor-Law Service, and if a large number of them could be induced to join the Association their subscriptions alone would be a valuable asset. But it also wanted the power to look after their interests and manage their affairs, and was afraid the College might interfere with this laudable object. A nursing section was therefore formed, and tremendous efforts are being made to induce the nurses to join it. The nursing section is only a branch of the parent Association, and the nurse is an officer of the Association first and foremost. Already the branch is said to have enrolled 900 nurses. What are their methods? I have lately had a letter from a superintendent-nurse of an unseparated infirmary of 500 beds, in which she says: "I have not registered yet, as so many people seem to think that Poor-Law nurses are not going to have any status if the College of Nursing scheme goes through, as they consider the Poor Law is not adequately represented in the Council." This letter shows how widespread are these misstatements about the College, and especially in the small infirmaries, that come so much more under the influence of the workhouse than the larger ones do. Many nurses in the small infirmaries are in a most difficult position, and are not always free agents. "Peaceful persuasion" is often very hard to resist, and often amounts to coercion.

What are the Real Facts?

The Association says that the Poor-Law nurse will have no status on the College. One speaker at the Liverpool meeting said that Poor-Law nurses would be placed in an unfair and untenable position in comparison with their colleagues who are in general hospitals. As a matter of fact, the nurse trained under the Poor Law is in exactly the same position on the College Register as the nurse trained in a general hospital. Her application is dealt with in exactly the same way, her vote will be of equal value. So far from having no status, or a lower status than the general hospital nurse, the Poor-Law nurse for the first time will be on an equality with her. Hitherto she has always had to show cause why she should be considered equal to the general hospital nurse. The College offers equal status and opportunity to all on the Register, and will not require the support of an outside Association. It is not Poor-Law nurses that are ignored by the College, but the Poor-Law Officers' Association; and rightly so, because the nurse registers as a nurse and not as a Poor-Law officer. The fact that the "Poor-Law Infirmary Matrons' Association" is solid against this movement of persuading nurses to join the Poor-Law Officers' Association shows that they realise the danger. They are the proper persons to look after the interests of their nurses, and there are three Poor-Law matrons on the present Council. The action of the Poor-Law Officers' Association is setting one class of nurses against another, and obscuring

(*Continued on p.* 486.)

**THE EDITOR'S LETTER-BOX**—(*Continued from page* 480.)

the fact that the College stands for the protection of the trained nurse against the half-trained, whether in Poor-Law or in general hospital. As registered nurses all will be equal, and eventually will all pass the same examination and receive the same certificate. It will then be seen that the infirmaries can turn out as efficient and intelligent nurses as the general hospitals.

A Campaign Throughout the Country.

Meetings should be held all over the country to counteract the Association's tactics, explain the true aims of the College, and to urge nurses to register at once. Poor-Law nursing seems to me to stand at the parting of the ways. Poor-Law nurses will either remain a class apart from other nurses and on a lower level by virtue of being associated in the public mind with the workhouse, or they will come to their true position in the College as registered nurses, on an equality with their fellow members, and be able to show by skilful work and by irreproachable conduct that they can hold their own with any branch of the profession. Now is the day of decision and the time for action. If trained Poor-Law nurses register at once, and form a strong body on the roll of the College, they can decide for themselves later on who they will have to represent them on the Council. But they will not expect to be treated as a class apart, but as honourable members of a great profession, of which the Royal College of Nursing is the outward and visible sign.—I am, Sir, yours faithfully,

A Matron.

